# Marine Microbial Gene Abundance and Community Composition in Response to Ocean Acidification and Elevated Temperature in Two Contrasting Coastal Marine Sediments

**DOI:** 10.3389/fmicb.2017.01599

**Published:** 2017-08-22

**Authors:** Ashleigh R. Currie, Karen Tait, Helen Parry, Beatriz de Francisco-Mora, Natalie Hicks, A. Mark Osborn, Steve Widdicombe, Henrik Stahl

**Affiliations:** ^1^Biogeochemistry and Earth Science, Scottish Association for Marine Science, Scottish Marine Institute Oban, United Kingdom; ^2^Plymouth Marine Laboratory Plymouth, United Kingdom; ^3^School of Biological Sciences, University of Hull Hull, United Kingdom; ^4^School of Science, Royal Melbourne Institute of Technology University, Bundoora VIC, Australia; ^5^Natural Science and Public Health, Zayed University Dubai, United Arab Emirates

**Keywords:** ocean acidification, ocean warming, muddy sediment, sandy sediment, microbial community, ammonia-oxidizing bacteria, denitrifying bacteria

## Abstract

Marine ecosystems are exposed to a range of human-induced climate stressors, in particular changing carbonate chemistry and elevated sea surface temperatures as a consequence of climate change. More research effort is needed to reduce uncertainties about the effects of global-scale warming and acidification for benthic microbial communities, which drive sedimentary biogeochemical cycles. In this research, mesocosm experiments were set up using muddy and sandy coastal sediments to investigate the independent and interactive effects of elevated carbon dioxide concentrations (750 ppm CO_2_) and elevated temperature (ambient +4°C) on the abundance of taxonomic and functional microbial genes. Specific quantitative PCR primers were used to target archaeal, bacterial, and cyanobacterial/chloroplast 16S rRNA in both sediment types. Nitrogen cycling genes archaeal and bacterial ammonia monooxygenase (*amoA*) and bacterial nitrite reductase (*nirS*) were specifically targeted to identify changes in microbial gene abundance and potential impacts on nitrogen cycling. In muddy sediment, microbial gene abundance, including *amoA* and *nirS* genes, increased under elevated temperature and reduced under elevated CO_2_ after 28 days, accompanied by shifts in community composition. In contrast, the combined stressor treatment showed a non-additive effect with lower microbial gene abundance throughout the experiment. The response of microbial communities in the sandy sediment was less pronounced, with the most noticeable response seen in the archaeal gene abundances in response to environmental stressors over time. 16S rRNA genes (*amoA* and *nirS*) were lower in abundance in the combined stressor treatments in sandy sediments. Our results indicated that marine benthic microorganisms, especially in muddy sediments, are susceptible to changes in ocean carbonate chemistry and seawater temperature, which ultimately may have an impact upon key benthic biogeochemical cycles.

## Introduction

Coastal zones are under substantial pressure from multiple human induced stressors (Halpern et al., 2008), including increased atmospheric carbon dioxide (*atm*CO_2_) levels. Since the start of the industrial revolution, the ocean has taken up approximately 25–30% of total human CO_2_ emissions (Sabine and Tanhua, 2010), resulting in perturbations to ocean carbonate chemistry and a reduction in pH. This “ocean acidification” (OA) affects the equilibrium of the ocean carbonate system, increasing bicarbonate (HCO_3_^-^) and hydrogen ions (H^+^) and decreasing pH and carbonate (CO_3_^2-^) concentration of the seawater (Zeebe and Wolf-Gladrow, 2001). This negative effect on the calcium carbonate (CaCO_3_) saturation state (Ω) of the seawater has varying effects on marine biota (Kroeker et al., 2010; and references therein). OA effects have been well studied in, calcifying organisms, which are particularly vulnerable to decreases in pH and Ω (e.g., coccolithophores: reviewed in Zondervan, 2007; coral: Langdon and Atkinson, 2005).

Increasing *atm*CO_2_ has also lead to elevated atmospheric and seawater temperatures, with global average seawater temperatures projected to increase between 1.8 and 4.0°C by the end of the 21st century (Solomon et al., 2007). Like OA, ocean warming has been shown to elicit various responses from marine bacteria and other microorganisms. For some heterotrophic bacteria, ocean warming is likely to increase bacterial growth (Piontek et al., 2009; Vázquez-Domínguez et al., 2012; Endres et al., 2014) whilst other studies have shown a significant reduction in size (Daufresne et al., 2009; Morán et al., 2015), also seen in other aquatic systems (Rasconi et al., 2015). Ocean warming can alter various ecosystem functions and associated services, influence changes to community structure (e.g., Hiscock et al., 2004; Mousing et al., 2014), and enhance carbon and nitrogen fluxes between phytoplankton and heterotrophic bacteria, indicating increased temperature may benefit mutualistic relationships of certain species (Arandia-Gorostidi et al., 2017).

Anthropogenic perturbations in the marine environment are known to alter microbially mediated biogeochemical cycles (Hutchins and Fu, 2017; Kitidis et al., 2017), such as the nitrogen (N) cycle (Vitousek et al., 1997; Galloway et al., 2004; Gruber and Galloway, 2008). The effects of OA on certain N-cycle pathways have recently been addressed, showing variable and inconsistent results. Nitrification (oxidation of NH_4_^+^ to NO_3_^-^) is the process most sensitive to pH change, due to the decline in the availability of ammonia (NH_3_) for nitrifying microorganisms (Suzuki et al., 1974). OA incubation experiments on planktonic microbes have shown a reduction in nitrification rates (Huesemann et al., 2002; Beman et al., 2011; Kitidis et al., 2011) and an increase in nitrogen fixation (Lomas et al., 2012), whilst contrasting studies have shown that a reduction in pH has no influence on planktonic microbial community composition and their associated biogeochemical processes (Newbold et al., 2012; Roy et al., 2013; Oliver et al., 2014). Reductions in the abundance of ammonia-oxidizing bacteria (AOB) and denitrifier transcripts have been seen in Arctic sediments exposed to elevated CO_2_ suggesting that the coupling of nitrification–denitrification may be affected (Tait et al., 2014). In contrast, Kitidis et al. (2011) and Watanabe et al. (2014) demonstrated there was no evidence to suggest sediment ammonia oxidation rates were inhibited at reduced pH levels. Mass budget modeling suggested OA can significantly reduce sediment nitrification rates (up to 94%) in two types of permeable sands, although the effects were more pronounced in pre-bloom conditions (Braeckman et al., 2014), and nutrient fluxes (and nitrification) under OA regimes can be mediated by a change in macrofaunal activity (Widdicombe and Needham, 2007; Widdicombe et al., 2009).

There is growing interest in the synergistic (or additive) effects of OA and increased temperature, as it is well documented that anthropogenically induced environmental changes tend not to occur in isolation (Crain et al., 2008; Halpern et al., 2008, 2015). However, the combination of inconsistent results and the limited number of experimental microbial studies makes it difficult to predict how nutrient cycling will be impacted by changes to more acidic and warmer seawater. Further interactions with additional environmental variables (such as nutrient availability) adds to the complexity in understanding stressor specific responses (Hutchins and Fu, 2017). This emphasizes the importance of more complex and integrated laboratory and field studies, which more closely mimic natural environments.

Integration of multiple stressors into experimental studies have indicated species-specific responses to different stressors within the same taxa. Fu et al. (2007) demonstrated the growth rate of the cyanobacteria *Synechococcus* increased under elevated temperature (ambient +4°C) but was not significantly higher in the elevated CO_2_ treatment. Within the same study, photosynthetic efficiency and carbon fixation were also shown to be enhanced when both drivers were combined for *Synechococcus*, but negative stressor effects were observed on the growth rate of cyanobacteria *Prochlorococcus*, suggesting warming or acidification could lead to shifts in community composition (Fu et al., 2007). Compositional shifts of a bacterioplankton population were identified when CO_2_ and temperature interacted, influencing the increase of certain microbial phylotypes (Lindh et al., 2013), and increased phytoplankton biomass has also been documented (Sommer et al., 2015).

Environmental changes occur synergistically and these interactions are likely to alter organisms response in a different way than exposure to an individual variable (Gunderson et al., 2016). Much of the biogeochemical cycling in the ocean is driven by microbial communities within sediments (Kitidis et al., 2017). However, different sediment types are infrequently compared, although the dynamics of cohesive and non-cohesive sediments are notably distinct in nutrient, carbon, and oxygen dynamics (Hicks et al., 2017a). Coastal and continental shelf sediments are areas of high carbon oxidation rates compared to deep sea sediments, and are key sites for biological carbon sequestration (Stahl et al., 2004; Burdige, 2006). Cohesive sediments (e.g., estuarine mud) have a high organic content and are mainly composed of small silt and clay grains, which form a highly active system both biologically and chemically (Black et al., 2002) and diffusive processes dominate the biogeochemistry (Hicks et al., 2017a). Non-cohesive sediments (e.g., sand) are permeable, by which advective flow is the major transport process of pore water moving solutes through and out of the sediment (Huettel et al., 2003). These contrasting sediments act differently in nature (e.g., biologically, physically and chemically) and it is likely that the response of their microbial communities will also differ when exposed to future climate conditions.

In the current study, a custom-built flume (mesocosm) facility was used to manipulate CO_2_ and temperature in order to test the impacts of seawater acidification and warming on microbial taxonomic marker- and nitrogen cycling-gene abundances, as well as on microbial community composition, in both muddy and sandy coastal sediments. Experimental treatment levels contained representatives of present day CO_2_ and temperature levels and of those expected by 2100 under a “business-as-usual” scenario (IPCC, 2007), and allow identification of individual and interactive effects of environmental stressors. These short-term experiments were designed to address the individual and interactive effects of reduced pH and increased seawater temperature on benthic microorganisms, which respond quicker to changing environmental parameters than higher trophic levels (Hicks et al., 2017b). Comparing how microorganisms in muddy and sandy sediments respond to elevated CO_2_ and temperature as both single and combined stressors at varying depths is a novel approach, and to our knowledge has not been fully explored in the repertoire of OA studies to date.

## Materials and Methods

### Experimental Design

Four environmental treatments were devised as follows: (1) control: 380 ppm CO_2_/12°C; (2) elevated temperature: 380 ppm CO_2_/16°C; (3) elevated CO_2_: 750 ppm CO_2_/12°C; (4) combined (elevated CO_2_ and elevated temperature): 750 ppm CO_2_/16°C (Supplementary Table S1). In each experimental run (referred to as “campaign”) a total of six flumes were used to contain the different sediment types (3 × mud; 3 × sand) with two of the four environmental treatments run at the same time within a campaign. Independent replication (*n* = 3) for four treatments were spread across four sampling dates: March 8, 2012; April 19, 2012; June 7, 2012; and July 19, 2012, due to the design of the flume facility. Any influence of seasonality, seen as differences between campaigns, on microbial gene abundance, relative sequence abundance and diversity indices was accounted for in the statistical analysis.

### Sediment Collection

Short-term (28 days) manipulation experiments were designed to investigate the response of microbial communities to OA and/or in combination with elevated temperature in muddy sediment (mean grain size = <63 μm) and sandy sediment (mean grain size = ∼200 μm) collected from the Eden Estuary near St Andrews (56°21.9N, 2°50.883W) and West Sands, St Andrews (56°22N, 2°49W), respectively. The top, oxic layer (determined visually by the sediment color change of the suboxic layer) of sediment was collected by hand. Macrofauna were removed from both sediment types using a 500 μm (muddy sediment) and a 1 mm (sandy sediment) mesh sieve in a seawater (UV treated; 1 μm filtered; salinity 35) bath and the sediment was left to settle for 24 h in large storage tanks to ensure retention of the fine particles.

After removing the supernatant and homogenizing the sediment, each sediment type was filled in three flumes (L 120 cm × H 30 cm × D 30 cm) with approximately 3.24 × 10^4^ cm^3^ of volume and a height of approximately 10 cm. Each of the six flumes was carefully filled with seawater (UV treated; 1 μm filtered; salinity 35) and left to settle in the flumes for 48 h. After 48 h, the supernatant was replaced again and the flumes were allowed to bubble with ambient air (380 ppm CO_2_) for 72 h before the acidified and elevated temperature treatments commenced. The overlying water in each flume was then replaced weekly, after sampling, to reduce the risk of the seawater becoming depleted in nutrients and to control for salinity changes.

**FIGURE 1:**
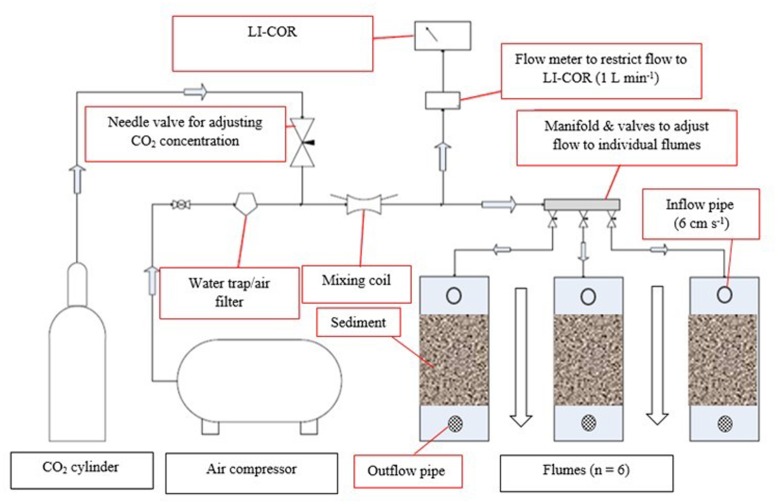
Experimental set up of the elevated CO_2_ system (750 ppm CO_2_). Dried ambient air from an external compressor is mixed with pure CO_2_ in a mixing coil and flowing (1 L min^-1^) into individual flumes and through a LI-COR 820 for continuous monitoring of concentration. The same set up was used for the ambient CO_2_ treatment (∼380 ppm CO_2_) for another three flumes, but without the addition of pure CO_2_. A recirculating flow of seawater via the inflow- and outflow-pipe was restricted to 6 cm s^-1^ and provided a unidirectional flow (indicated by a black-outlined arrow).

Recirculating pumps (Pisces SC050, Pisces Engineering, United Kingdom) provided a unidirectional and even flow (6 cm s^-1^) over the sediment surface in each flume, reflecting average *in situ* water movement. Temperature was manipulated individually in each flume using submerged 500W titanium aquarium heaters with a digital control unit (Aqua Medic T-meter, Germany) and the CO_2_ concentration of the injected gas was continuously monitored by gas analyzers (LI-COR^®^ Biosciences, Inc., United States). Dried ambient air (CO_2_ = 380 ppm) or air-enriched CO_2_ mix (CO_2_ = 750 ppm) were introduced into the overlying water of the flumes at a rate of 1 L min^-1^ using submerged bubble stones, from an external air compressor and a cylinder of compressed CO_2_ (**Figure 1**).

Artificial light was provided by 2 × 80 W neon lamps (T5 Biolight, Osram Licht AG, Germany) that were placed parallel above each fume tank to reproduce *in situ* light conditions. The light intensity was set to 40–50 μE m^-2^ and operated on a 12 h day/night cycle for the duration of the experiment. Daily measurements were taken for temperature, pH, and salinity in the overlying water and weekly water samples were taken for dissolved inorganic carbon (DIC) and total alkalinity (A_T_).

### Carbonate System Analysis

Weekly water samples were taken in both light and dark conditions for A_T_ (30 mL) and DIC (12 mL), and were poisoned with 50 μL of a saturated mercuric chloride (HgCl_2_) solution. Samples were stored in acid-washed and thoroughly rinsed gas tight glass bottles and placed in a fridge (4°C) until analysis. A_T_ samples were analyzed using an automatic potentiometric titrator (888 Titrando, Metrohm, Switzerland) with Tiamo^®^ V 2.1 software. A three-point calibration was performed using buffer solutions pH 4, 7, and 9 (Metrohm UK Ltd.) before analysis. The precise volume of acid added was plotted against pH to form a curve, which was then logged to produce a straight line, and the gradient was calculated to obtain A_T_ (Dickson et al., 2007). Certified CO_2_ reference material (Andrew G. Dickson, Scripps Institution of Oceanography, CA, United States) was used to monitor the accuracy of the titrator (Dickson et al., 2003).

DIC was determined using a CM140 Total Inorganic Carbon Analyzer (UIC Inc, United States) performed by the method of Dickson et al. (2007). Calibration was carried out by running blanks to determine the carrier gas carbon content followed by seawater standards of known concentration to ascertain precision and accuracy within ±0.01 mmol l^-1^. Prior to sample analysis a standard solution of sodium bicarbonate (NaHCO_3_), made to known concentrations, was run until a precision of 0.03% deviation was achieved from three consecutive samples. Routine checks on accuracy were made using commercially available IAPSO seawater standards.

### Water Column Nutrients

Filtered water samples (0.45 μm pore size) were taken weekly with a sterile 50 mL plastic syringe and dispensed into clean 45 mL centrifuge tubes and stored at -20°C. Prior to analysis, samples were defrosted and gently inverted to ensure the water was evenly mixed. Triplicate samples were analyzed for ammonium (NH_4_^+^), phosphate (PO_4_^+^), nitrite + nitrate (NO_2_^-^ + NO_3_^-^) (herein referred to as NO_X_). Nutrient analysis was performed using a Lachat 8500 Flow Injection autoanalyzer (Lachat Instruments) following Grasshoff et al. (1999) and recommended instrument manufacturers’ methods [ammonia (Liao, 2008), phosphate (Egan, 2008), and nitrate + nitrite (Diamond, 2008)]. Standard concentration range was adjusted for sample concentrations and made using OSIL low nutrient seawater (North Atlantic salinity = 35 psu) for standard preparation. The machine was calibrated using laboratory made nutrient standards before and after sample analysis to ascertain precision and accuracy within ±2 and ±3% of the true value, respectively. The methods are based on classical wet chemical reactions and using 1 cm path-length flow-cell spectrophotometry for detection.

### Sediment Sampling and DNA Extraction

Sediment for microbial analysis was collected using syringe core samples (10 mL syringe). Four samples were taken from each flume at three time points during the experiment (days 0, 7, and 28), giving a total of 12 measurements from each environmental treatment. Syringe core samples were taken to a depth of ∼5 cm and immediately frozen (-20°C). The surface sediment samples were split into different depths (muddy: upper 0–0.5 cm; bottom 0.5–2.5 cm and sandy: 0–1 cm). The sample depths were based on oxygen microprofile measurements (not presented here), which indicated the maximum oxygen penetration depth was ∼0.4 cm in the muddy sediment and ∼1 cm in the sandy sediment. Based on the information gained from the oxygen penetration depth data, we assumed that AOB/archaea were most active between 0 and 0.5 cm in the muddy sediment, and denitrifying bacteria active below this point, in the suboxic layer. Although denitrifying bacteria exist in the upper oxygenated layer within anoxic/suboxic “micro-niches,” this surface layer was targeted as a higher density area. In the sandy sediment, analyses were run on all genes at one depth layer as it was unlikely gene abundances would be above the detection limit for quantitative PCR (q-PCR) below this depth.

Prior to DNA extraction, each sediment sample was homogenized by stirring with a sterile metal spatula. For muddy sediments, DNA was extracted from 0.25 g of each sediment sample using MoBio Powersoil^®^ DNA extraction kit (MoBio, Carlsbad, United States) according to the manufacturer’s instructions. For sandy sediments, a modified protocol using 0.5 g sediment was used. First, 200 μL bead solution was removed from the bead tube and replaced with 200 μL of phenol:chloroform:isoamyl alcohol (pH 8) (25:24:1) (Sigma, Gillingham, United Kingdom). Solution C1 (60 μL) was then added and the bead tube vortexed at maximum speed for 10 min. Following centrifugation for 1 min at 10,000 *g*, the upper aqueous layer was removed, placed in a new tube, 100 μL Solution C2 and 100 μL Solution C3 added, mixed and incubated on ice for 5 min. The tube was centrifuged for 1 min at 10,000 *g* and the supernatant removed to a new tube. The remaining protocol then followed the manufacturer’s instructions. Quantification of the extracted DNA was carried out using the Quant-iT^TM^ PicoGreen^®^ dsDNA Assay Kit (Invitrogen, Thermo Fisher Scientific, Basingstoke, United Kingdom) according to the manufacturer’s instructions.

### Quantitative PCR

q-PCR analysis was conducted using an ABI 7000 sequence detection system (Applied Biosystems, Foster City, United States) and Quantifast SYBR^®^ Green PCR Kit (Qiagen^®^) using a selection of primers to target taxonomic and nitrogen cycling genes (Supplementary Table S2). PCR primers specific for archaea, bacteria, and cyanobacterial/chloroplast 16S rRNA genes were used to quantify the abundance of archaea, bacteria, and microphytobenthos. For nitrogen cycling genes, PCR primers specific for archaeal and bacterial ammonia monooxygenase (*amoA*) and bacterial nitrite reductase (*nirS*) genes were utilized. For each gene, triplicate assays were performed using standard curves ranging from 10^2^ to 10^8^ amplicons μL^-1^ DNA. For each primer, standard curves were established using cloned sequences and nucleic acids were quantified using a NanoDrop spectrophotometer (NanoDrop Technologies, DE, United States). Gene numbers were quantified by comparison to standard curves using the ABI Prism 7000 detection software. Automatic analysis settings were used to determine the threshold cycle (C_T_) values and baselines settings. The no-template controls were below the C_T_ threshold in all experiments. For each standard curve, the slope, *y* intercept, co-efficient of determination (*r*^2^) and the efficiency of amplification were determined (Supplementary Table S3). The abundance of bacterial and archaeal 16S rRNA and *nirS* genes were quantified in both muddy sediment layers, but cyanobacterial/chloroplast 16S rRNA and *amoA* genes were only quantified in the upper sediment layer. Each qPCR assay was conducted twice and the numbers of genes g^-1^ sediment averaged.

### Analyses of 16S rRNA Gene Sequences

The relative abundance and composition of 16S rRNA genes in the muddy (0–0.5 cm) samples was determined using 16S rRNA tagged Illumina MiSeq. The V1–V3 region of 16S rRNA was amplified using the PCR primers 27F (AGRGTTTGATCMTGGCTCAG; Vergin et al., 1998) and 519Rmod (GTNTTACNGCGGCKGCTG; Andreotti et al., 2011). The 50 μL reaction volume contained 1 μL DNA, 10× PCR buffer (Qiagen, Manchester, United Kingdom), 2 mM MgCl_2_, 0.2 mM dNTPs, 1.5 U of Taq DNA polymerase (Qiagen, Manchester, United Kingdom), and 0.5 μM of forward and reverse primers. PCRs were initially denatured for 3 min at 94°C, followed by 25 cycles of 94°C for 30 s; primer annealing at 57°C for 45 s, and elongation at 72°C for 60 s. A final elongation step was performed at 69°C for 5 min. Each sediment sample was amplified in triplicate, and the triplicates pooled and cleaned using the QIAquick PCR purification kit (Qiagen, Manchester, United Kingdom), and sent to MR DNA^1^ (TX, United States). PCR products were then subjected to a further five PCR cycles using primer sets modified with multiplexing identifier adaptors for barcode tagging, thereby allowing for post-sequencing separation of the samples. Following PCR, all amplicon products from different samples were mixed in equal concentrations and purified using the Agencourt AMPure XP Purification System (Beckman Coulter, Bromley, United Kingdom). The pooled and purified PCR product was used to prepare DNA library by following the Illumina TruSeq DNA library preparation protocol. Sequencing was performed on a MiSeq following the manufacturer’s guidelines. Sequence data were processed using a proprietary analysis pipeline (MR DNA, TX, United States) as follows: sequences were de-multiplexed, depleted of barcodes and primers, sequences <150 bp or with ambiguous base calls and with homopolymer runs exceeding 6 bp removed, denoised, operational taxonomic units (OTUs) generated (at 97% similarity) and chimeras removed. Final OTUs were taxonomically classified using BLASTn against a curated GreenGenes database (DeSantis et al., 2006). All unclassified OTUs and those containing less than five sequences per OTU were removed. To allow comparison between samples, all samples were sub-sampled to the lowest value: 11495. The sequence data and associated metadata is available via http://www.ncbi.nlm.nih.gov/bioproject/378259 and http://www.ncbi.nlm.nih.gov/biosample/6481972. All data from this study can be accessed on the BODC website^2^.

### Statistical Analyses

The carbonate chemistry was analyzed using an ANOVA for each sediment type and each carbonate chemistry parameter (A_T_ and DIC) in RStudio 1.0.136 (RStudio Team, 2016).

Statistical analyses for gene abundance (g^-1^ sediment) were carried out using the statistical programming software R version 3.1.2 (R Development Core Team, 2015), with each sediment type analyzed separately. The gene abundance results were analyzed using linear-mixed effects models (package lm4, version 1.1.17; Bates et al., 2014) to assess the effect of CO_2_, temperature, day (time point), and the interaction on the abundance of various microbial genes. As the data are nested (i.e., there were four repeated measurements take on day 0, 7, and 28 for each treatment replicate), using a linear mixed-effects model imposes a dependency structure for all gene abundance values to account for the spatial correlation. The dependency structure in the gene abundance values would produce Type I errors and biased parameter estimates if a linear regression model was used instead. Mixed-effects models account for the variability of gene abundance in each flume by calculating a random slope and a random intercept, thus taking into account the differences in abundance that may have been influenced by seasonality (campaign). All random variables were assumed to be normally distributed with a zero mean.

For model selection, a restricted maximum likelihood (REML) estimation procedure (West et al., 2006) was applied to establish the contribution of the parameters CO_2_, temperature and day. The response variable (gene abundance) was normalized using a square-root or log_10_ transformation prior to analysis with a linear mixed-effects model. Non-significant terms were removed by backward manual selection and excluded from further analyses. The *p*-values were estimated from the parameter’s *t*-values and the degrees of freedom. In order to determine the accuracy of the model estimations, model fit was assessed by examining the distribution, bias and precision of residuals using Cook’s distance and QQ-plots (Zuur et al., 2009).

The package nlme: linear and non-linear mixed effects models (Pinheiro et al., 2016) was used to statistically analyze community composition data (phylum and class) and alpha diversity in R version 3.1-127 (R Development Core Team, 2015). A linear mixed model was run using REML for relative sequence abundance (%) or diversity indices (i.e., species richness, Pielou evenness, and Shannon–Wiener), using treatment and day (time point) as fixed effects and campaign as a random effect. The “best fit” models were assessed using Akaike’s Information Criterion and the accuracy of the models was determined as outlined above. Where possible, a Tukey’s test was run for all phyla and classes to establish all possible pairwise comparisons among means (Hothorn et al., 2008).

## Results

There were clear differences between sediment type, from the carbonate chemistry and nutrient dynamics to the microbial community response.

### Carbonate Chemistry

**FIGURE 2:**
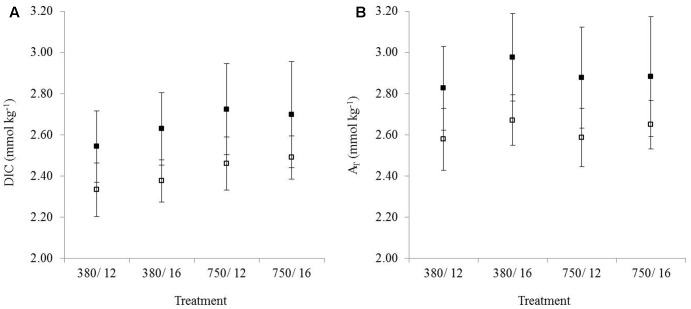
The mean (*n* = 3) concentration of **(A)** DIC (dissolved inorganic carbon) and **(B)** A_T_ (total alkalinity) from the control and treatment flumes. Error bars represent the standard deviation. Samples were taken from the overlying water in the daytime for muddy sediment (solid black squares) and sandy sediment (white squares). Treatments on the *x*-axis represent environmental conditions during the experiment.

Observations showed that DIC (**Figure 2A**) and A_T_ (**Figure 2B**) were consistently higher in the muddy sediment compared to the sandy sediment across all treatments, and sediment was highly significant for both A_T_ (*p* < 0.001) and DIC (*p* < 0.001). Despite this, the overall trends for DIC and A_T_ were similar between the sediment types. DIC was significantly affected by only CO_2_ (*p* < 0.001), with no temperature or combined stressors effects (**Figure 2A**). In contrast, A_T_ was significantly influenced by temperature (*p* < 0.001), and the interaction between CO_2_ and temperature was significant (*p* = 0.028), showing that the combined stressor effect was significant even though CO_2_ was not significant as a single stressor (**Figure 2B**).

### Nutrients in the Overlying Water

The nutrient dynamics were strongly influenced by sediment type. The average nutrient concentrations (*n* = 3) recorded weekly in the overlying water for both sediment types are provided in **Table 1**. Here we only present the nutrient concentrations for the measurements taken in the light period, as a *t*-test showed that there was no significant difference between the means within each treatment taken in the light and the dark period (*p* > 0.5).

**Table 1:** Mean nutrient concentrations (±standard deviation of mean) for ammonium (NH_4_^+^), nitrite + nitrate (NO_X_), and phosphate (PO_4_^3-^) recorded in the overlying water taken weekly in each environmental treatment for muddy and sandy sediments.

			Mud	Sand	Mud	Sand	Mud	Sand
			
CO_2_	Temperature	Week	NH_4_^+^	NH_4_^+^	(NO_X_)	(NO_X_)	PO_4_^3-^	PO_4_^3-^
(ppm)	(°C)		(μmol dm^-3^)	(μmol dm^-3^)	(μmol dm^-3^)	(μmol dm^-3^)	(μmol dm^-3^)	(μmol dm^-3^)
		1	10.02 ± 8.05	10.68 ± 6.46	10.41 ± 7.14	2.99 ± 3.62	0.13 ± 0.05	0.23 ± 0.17
380	12	2	18.19 ± 13.09	4.73 ± 5.15	24.98 ± 22.17	0.45 ± 0.52	0.16 ± 0.14	0.12 ± 0.16
		3	5.57 ± 8.06	5.23 ± 7.53	21.22 ± 10.61	0.72 ± 0.78	0.04 ± 0.03	0.12 ± 0.08
		4	6.47 ± 10.42	7.54 ± 10.23	27.52 ± 24.38	0.40 ± 0.32	0.05 ± 0.04	0.07 ± 0.04
		1	25.35 ± 16.42	8.28 ± 8.00	12.63 ± 6.60	21.40 ± 23.62	0.09 ± 0.03	0.94 ± 0.54
380	16	2	28.33 ± 45.25	6.04 ± 3.66	38.08 ± 10.30	15.52 ± 15.09	0.19 ± 0.05	0.71 ± 0.64
		3	3.67 ± 4.99	2.20 ± 3.18	49.73 ± 29.70	1.26 ± 2.70	0.26 ± 0.10	0.88 ± 0.80
		4	1.65 ± 3.47	1.43 ± 2.64	43.00 ± 30.98	2.39 ± 3.60	0.24 ± 0.17	1.15 ± 0.67
		1	15.47 ± 15.14	10.30 ± 8.76	6.03 ± 3.94	14.10 ± 20.05	0.07 ± 0.04	0.87 ± 0.51
750	12	2	18.58 ± 15.95	8.08 ± 8.10	22.79 ± 6.65	4.80 ± 5.64	0.10 ± 0.06	0.36 ± 0.54
		3	22.57 ± 16.70	9.00 ± 9.23	37.02 ± 15.70	1.02 ± 2.11	0.08 ± 0.08	0.47 ± 0.69
		4	4.14 ± 5.33	6.86 ± 10.58	39.47 ± 17.97	1.37 ± 2.12	0.09 ± 0.05	0.92 ± 0.59
		1	7.58 ± 8.77	5.46 ± 6.43	14.48 ± 14.33	1.09 ± 1.08	0.20 ± 0.11	0.18 ± 0.13
750	16	2	13.92 ± 23.29	2.58 ± 5.23	20.92 ± 22.04	0.40 ± 0.63	0.12 ± 0.14	0.28 ± 0.48
		3	5.26 ± 7.72	4.98 ± 8.24	31.25 ± 33.39	0.73 ± 0.42	0.04 ± 0.03	0.06 ± 0.05
		4	2.32 ± 2.94	2.94 ± 3.12	34.34 ± 39.46	0.59 ± 0.39	0.08 ± 0.10	0.08 ± 0.06

*Observations are shown for the light period only, as the means in the light and dark phase did not show any significant difference*.

In the muddy sediment, NH_4_^+^ concentration in the overlying water increased in the second week then decreased over time. In contrast, NO_X_ showed the opposite pattern with increasing NOx over time as NH_4_^+^ is oxidized to NO_X_. There are no obvious trends for PO_4_^3-^ concentration, possibly due to PO_4_^3-^ being more readily absorbed to Fe/ FeS in the muddy sediment in comparison to the sandy sediment. In general, nutrient concentrations were higher in the muddy compared to the sandy sediments, in which the highest concentrations of all three nutrients are in the single stressor elevated temperature treatment. The lower nutrient concentrations for NH_4_^+^ and NOx from the sandy sediment incubations illustrate how the advective flow in permeable sediments prevents higher levels of organic matter or nutrients from accumulating. There were no clear trends for NH_4_^+^ over time, consistent with typical permeable, well oxygenated sediments. NO_X_ appeared to decrease over time compared to the muddy sediment, where it increased over time. PO_4_^3-^ showed higher values in the permeable sediments as there is less Fe/FeS compounds that PO_4_^3-^ will absorb to. There were no clear trends in temporal development, although NO_X_ concentrations were higher in the elevated temperature treatment and the start of the elevated CO_2_ treatment, but there was no difference between the control and combined stressor treatments.

### q-PCR Quantification of Gene Copy Numbers in Muddy Sediment Samples

**FIGURE 3:**
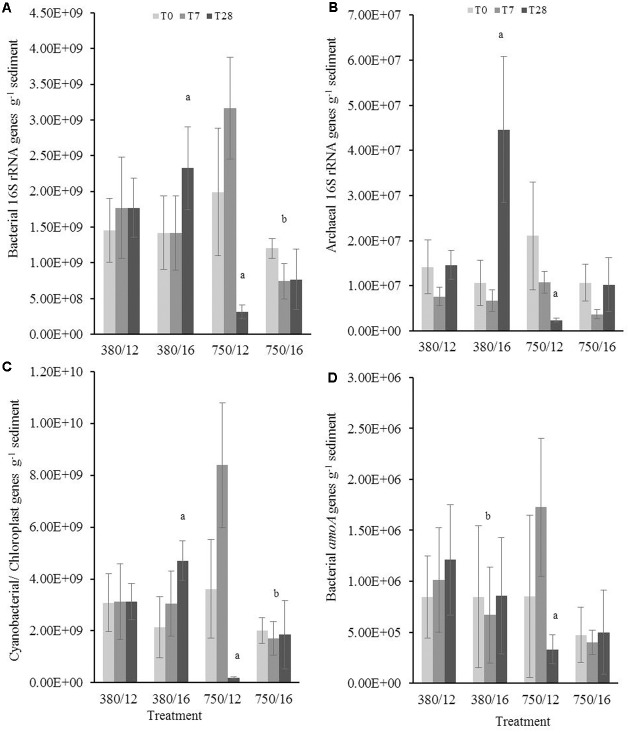
The independent and interactive effects of CO_2_ and or temperature on the abundance (g^-1^ sediment) of **(A)** bacterial 16S rRNA, **(B)** archaeal 16S rRNA, **(C)** cyanobacterial/chloroplast 16S rRNA, and **(D)** bacterial *amoA* genes in the upper layer (0–0.5 cm) of muddy sediment. Four syringe cores were taken at each time point (T0, light gray; T7, dark gray; and T28, black) from each environmental treatment (*x*-axis). Significant effects from the model output are indicated by the letter a (where there is an interaction with day); b (interaction between elevated CO_2_ and elevated temperature). Error bars represent standard deviation of repeated measurements (*n* = 4) from treatment replicates (*n* = 3).

Results from the linear mixed-effects model (Supplementary Table S4) for the upper sediment samples (0–0.5 cm) showed that bacterial 16S rRNA gene abundance (**Figure 3A**) increased significantly in response to elevated temperature at day 28 (*t* = 2.37, *p* = 0.01). In contrast, at day 28 in the elevated CO_2_ treatment samples, the gene abundances of bacterial 16S rRNA genes significantly decreased (*t* = -4.62, *p* < 0.0001). The combined effects of elevated CO_2_ and elevated temperature on bacterial 16S rRNA gene abundance showed that there was an interaction between the two factors with gene abundance being significantly lower (*t* = -2.47, *p* = 0.01). Gene abundance for archaeal 16S rRNA (**Figure 3B**) was significantly higher at day 28 when exposed to elevated temperature (*t* = 0.56, *p* < 0.001) and significantly lower in the elevated CO_2_ treatment (*t* = -0.94, *p* < 0.0001). The gene abundance of photosynthetic autotrophic microorganisms (**Figure 3C**) were found to increase at day 28 of the elevated temperature treatment (*t* = 2.99, *p* = 0.002) and decrease in the elevated CO_2_ treatment (*t* = -4.285, *p* < 0.0001). Cyanobacterial/chloroplast 16S rRNA genes were lower when CO_2_ and temperature were elevated, suggesting that the interactive effects have a significant influence on the abundance of primary producers (*t* = -2.02, *p* = 0.04). Elevated temperature was identified as a main effect in the model, showing that the gene abundance of AOB (*amoA*) was significantly lower when the sediment was exposed to a temperature of 16°C (**Figure 3D**; *t* = -0.34, *p* < 0.0001). The model further indicated a significant effect of elevated CO_2_ at day 28 where we see a lower abundance of bacterial *amoA* (*t* = -2.11, *p* = 0.03).

**FIGURE 4:**
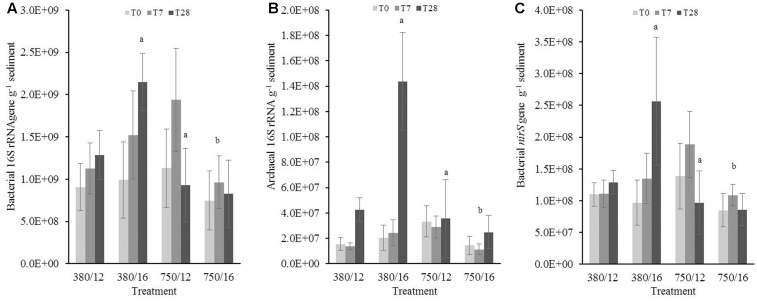
The effects of CO_2_ and/or temperature on the abundance (g^-1^ sediment) of **(A)** bacterial 16S rRNA, **(B)** archaeal 16S rRNA, and **(C)** bacterial *nirS* genes in the bottom layer (0.5–2.5 cm) of muddy sediment. Four syringe cores were taken at each time point (T0, light gray; T7, dark gray; and T28, black) from each environmental treatment (*x*-axis). Significant effects from the model output are indicated by the letter **a** (where there is an interaction with day); **b** (interaction between elevated CO_2_ and elevated temperature). Error bars represent standard deviation of repeated measurements (*n* = 4) from treatment replicates (*n* = 3).

In the deeper muddy sediment layer (0.5–2.5 cm), the abundance of bacterial 16S rRNA genes (**Figure 4A**) at day 28 significantly increased (*t* = 2.47, *p* = 0.01) at elevated temperature and was significantly lower in the elevated CO_2_ treatment (-3.87, *p* < 0.001). When the sediment was exposed to the interactive effects of elevated CO_2_ and elevated temperature, the bacterial gene abundance was significantly lower (*t* = -3.89, *p* < 0.0001). Elevated CO_2_ was shown to be a significant main effect driving the abundance of bacterial and archaeal 16S rRNA genes in the lower sediment layer. Archaeal 16S rRNA gene abundance (**Figure 4B**) was significantly higher at day 28 in the elevated temperature treatment (*t* = 4.08, *p* < 0.0001) and was significantly lower at day 28 in the elevated CO_2_ treatment (*t* = -6.27, *p* < 0.0001). There was a significant interaction between the two fixed factors, where we saw a substantially lower archaeal 16S rRNA gene abundance (*t* = -5.56, *p* < 0.0001). The model indicated there was a main effect of both elevated CO_2_ and temperature on the nitrate reducing bacteria gene (*nirS*) abundance. At day 28, bacterial *nirS* gene abundance was shown to be significantly higher (*t* = 3.79, *p* = 0.001) when temperature was elevated to 16°C (**Figure 4C**). In contrast, the results indicated a significantly lower gene abundance at day 28 in the elevated CO_2_ treatment (*t* = -4.87, *p* < 0.0001).

Zero or very low archaeal *amoA* genes were present within the muddy sediments (0–10 copies g^-1^ sediment) and therefore will not be discussed further. This result was confirmed using primers from (Francis et al., 2005) and Wuchter et al. (2006) (Supplementary Table S2).

### q-PCR Quantification of Gene Copy Numbers in Sandy Sediment Samples

**FIGURE 5:**
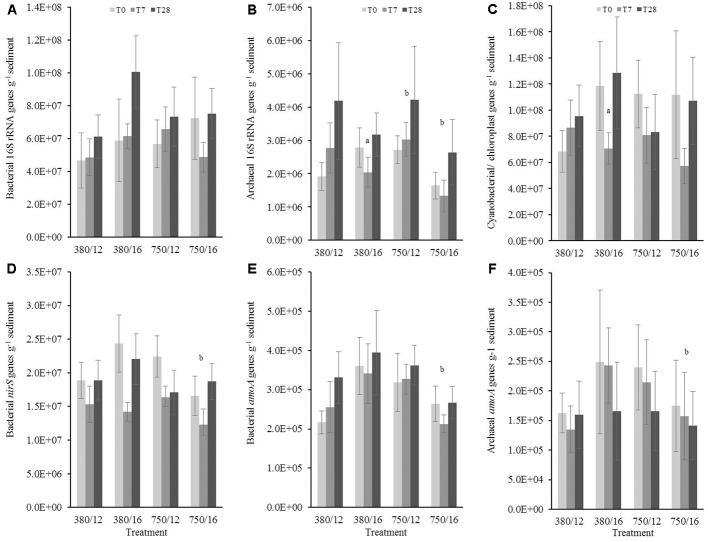
The effects of CO_2_ and/or temperature on the abundance (g^-1^ sediment) of **(A)** bacterial 16S rRNA, **(B)** archaeal 16S rRNA, **(C)** cyanobacterial/chloroplast 16S rRNA, **(D)** bacterial *nirS*, **(E)** bacterial *amoA* genes, and **(F)** archaeal *amoA* genes in the top layer (0–1 cm) of sandy sediment. Four syringe cores were taken at each time point (T0, light gray; T7, dark gray; and T28, black) from each environmental treatment (*x*-axis). Significant effects from the model output are indicated by the letter **a** (where there is an interaction with day); **b** (interaction between elevated CO_2_ and elevated temperature). Error bars represent standard deviation of repeated measurements (*n* = 4) from treatment replicates (*n* = 3).

Bacterial 16S rRNA gene copies did not show a significant increase or decrease (**Figure 5**) in response to the independent and interactive variables. Any significant changes were related to day 28, suggesting there was not a strong treatment effect on bacterial 16S rRNA gene abundance, although there were clear differences at 28 days for the elevated temperature and 7 days for the combined stressor treatment (**Figure 5A**). The results from archaeal 16S rRNA gene copies showed that there was a much clearer response to the treatments (**Figure 5B**). The model results indicated elevated CO_2_ as a main effect, which was marginally influencing archaeal 16S gene abundance (*t* = 1.94, *p* = 0.05). There was an interaction at day 7 in the elevated temperature treatment where gene abundance was significantly lower (*t* = -2.37, *p* = 0.01). Archaeal gene abundance was strongly influenced by the interaction between elevated CO_2_ and elevated temperature where abundance was noticeably lower (*t* = -4.26, *p* < 0.0001). Photosynthetic autotrophic microorganisms (**Figure 5C**) in the sandy sediment were not impacted greatly by the different treatments, the only significant response was due to elevated temperature within only 7 days (*t* = -2.02, *p* = 0.04), and this temporal trend was also seen in the combined stressor treatment.

There was a strong single stressor effect on bacterial *nirS*, bacterial *amoA*, and archaeal *amoA* gene abundance, showing higher initial abundance for all three genes in both elevated temperature and elevated CO_2_, but no significant difference in the combined stressor treatment (see **Figures 5D–F**). The results from the model suggested that the abundance of archaeal *amoA* was influenced by elevated temperature, where gene abundance also showed a slight significant increase to the temperature as a single stressor (*t* = 0.13, *p* = 0.05). In contrast to the muddy sediment, the observed results for gene abundance in sandy sediments did not show strong similarities between genes.

### Microbial Community Composition in Muddy Sediment Samples

The impacts of elevated CO_2_ and temperature on gene abundance were more marked in the muddy than sandy sediments, thus the effects of elevated CO_2_ and temperature on the composition of the microbial community in the upper layer were therefore investigated in the muddy sediment using in-depth 16S rRNA gene amplicon sequence analysis.

**FIGURE 6:**
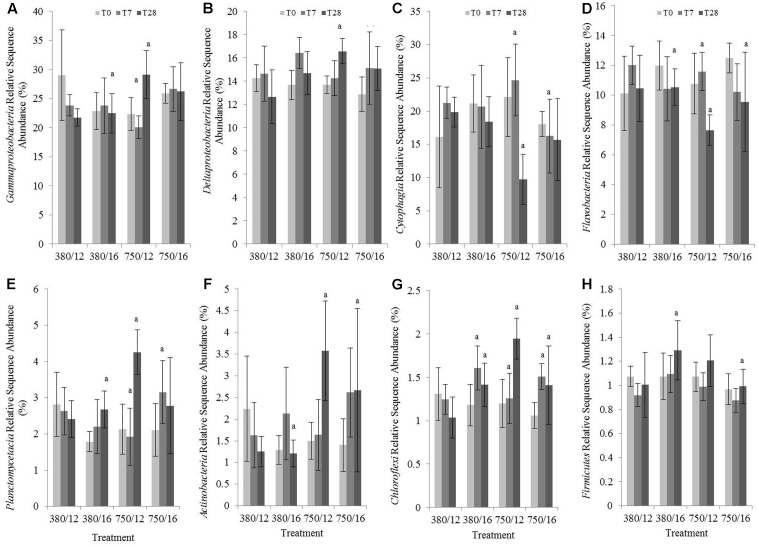
The effects of CO_2_ and/or temperature on the relative sequence abundance (%) of **(A)** Gammaproteobacteria, **(B)** Deltaproteobacteria, **(C)** Cytophagia, **(D)** Flavobacteria, **(E)** Planctomycetacia, **(F)** Actinobacteria, **(G)** Chloroflexi, **(H)** Firmicutes in muddy sediment. Four syringe cores were taken at each time point (T0, light gray; T7, dark gray; and T28, black) from each environmental treatment (*x*-axis). Significant effects from the model output are indicated by the letter a (where there is an interaction with day); b (interaction between elevated CO_2_ and elevated temperature). Error bars represent standard deviation of repeated measurements (*n* = 4) from treatment replicates (*n* = 3).

Relative abundance sequence data was averaged per treatment for the major phyla and classes present. The community was dominated by members of the Proteobacteria, in particular the Gammaproteobacteria (relative sequence abundance of 24.5%), Deltaproteobacteria (14.5%), Alphaproteobacteria (6.6%), and members of the Bacteroidetes Cytophagia (18.7%), Flavobacteria (10.7 %). The impact of CO_2_ was more marked than temperature, with several phyla and classes showing significant changes to relative sequence abundance within the elevated CO_2_ treatments (**Figure 6** and Supplementary Table S5). At day 28 under elevated CO_2_ (single stressor treatment), significant increases to the relative abundance were evident for the class Gammaproteobacteria (**Figure 6A**), class Deltaproteobacteria (**Figure 6B**), class Planctomycetacia (**Figure 6E**), phylum Actinobacteria (**Figure 6F**), and phylum Chloroflexi (**Figure 6G**), and decreases to the relative abundance of the Bacteroidetes classes Cytophagia (**Figure 6C**) and Flavobacteria (**Figure 6D**). For the Gammaproteobacteria, there was a significant increase in abundance between day 7 and day 28 in the independent elevated CO_2_ treatment (Tukey’s test *z* = 4.793; *p* < 0.01), and an increase in abundance within the elevated CO_2_ treatment (750 ppm CO_2_/12°C) when compared to when elevated temperature was elevated individually (380 ppm CO_2_/16°C) at day 28. This was predominantly due to an increase to the relative abundance of the orders Alteromonadales, Chromatiales, Acidithiobacillales, Pseudomonadales, and Oceanospirillales within the elevated CO_2_ treatment. Modest increases to the relative abundance of the orders Myxococcales and Desulfobacterales were responsible for the increased relative abundance of Deltaproteobacteria within the elevated CO_2_ treatment after 28 days incubation (*z* = 3.536; *p* = 0.01). Decreases to the relative abundance of Cytophagia in the elevated CO_2_ treatment at day 28 (*z* = -5.025, *p* < 0.01), and at day 7 in the elevated CO_2_ treatment compared to day 7 the interactive variable treatment (*z* = -3.413, *p* = 0.03), and at day 28 in the elevated CO_2_ treatment compared to day 28 in the elevated temperature treatment (*z* = -3.526, *p* = 0.02) could be traced to decreases to the genus *Hymenobacter*. A significant decrease in the relative abundance of Flavobacteria in the elevated CO_2_ treatment at day 28 (*z* = -3.509, *p* = 0.01) was observed, and the large decrease from day 7 to day 28 in CO_2_ single stressor treatment (*z* = -4.398, *p* < 0.01) were due to decreases within the Flavobacteriaceae of *Owenweeksia*, *Maritimimonas*, *Sufflavibacter*, and *Ulvibacter*. The relative abundance of Flavobacteria was also significantly lower in the combined variable treatment at day 28 (*z* = -3.313, *p* = 0.03). Of the dominant phyla, the only taxa showing significant differences in relative abundance in response to temperature were the Firmicutes (**Figure 6H**). Relative abundance of Firmicutes was significantly greater in the elevated temperature treatment at day 28 (*z* = 3.456, *p* = 0.02) and significantly lower at day 28 in the combined stressor treatment, particularly when compared to day 28 in the elevated temperature treatment (*z* = -3.613, *p* = 0.01). These observed changes were driven by increases in the relative abundance of Clostridiales within Firmicutes.

No differences in measurements of alpha diversity (species richness, evenness, or Shannon diversity) between treatments were detected (Supplementary Figure S1).

## Discussion

The current study demonstrates the response of microbial communities in muddy and sandy surface sediments exposed to elevated CO_2_ (750 ppm) and/or elevated temperature (16°C), mimicking short-term climate change perturbations. As the sediments used in this study were devoid of macrofauna any changes in microbial gene abundance and community composition were attributed to the various environmental treatments.

### Carbonate Chemistry in the Overlying Water

Surface A_T_ in the open ocean is on average 2.3 mmol kg^-1^ (Lee et al., 2006) and DIC is around 2.05–2.1 mmol kg^-1^ (Feely et al., 2001). In the current study, A_T_ and DIC levels were considerably higher in the overlying water of both sediment types. This deviation from the open ocean average is expected in coastal and estuarine habitats, which experience daily fluctuations with tidal changes (e.g., salinity). The muddy sediment A_T_ and DIC (∼2.8 and 2.6 mmol kg^-1^, respectively) were consistently higher than the sandy sediments (∼2.6 and 2.35 mmol kg^-1^, respectively). Water samples for DIC and A_T_ were taken <10 cm above the sediment in the overlying water, where concentrations are typically much higher due to intense diagenetic activity within coastal sediments (Stahl et al., 2004). The concentration gradient across the sediment–water interface will drive effluxes of DIC and A_T_ from the sediment into the overlying water, resulting in elevated concentrations of these constituents in the bottom water (Stahl et al., 2004).

Higher DIC concentrations in treatments with elevated CO_2_ are likely due to the invasion of excess CO_2_ in the overlying water leading to an increase in DIC but not A_T_ (Zeebe and Wolf-Gladrow, 2001). A_T_ concentration is notably higher in the elevated temperature treatment, which is likely explained by stimulated respiration and possibly CaCO_3_ dissolution. As respiration is stimulated CO_2_ production increases locally, concurrently reducing pH in the sediment which would lead to CaCO_3_ dissolution (Glud, 2008). Dissolution of one mole of CaCO_3_ results in an increase in alkalinity by two moles, as CO_3_^2-^ is equal to two A_T_ equivalents (CaCO_3_ ⇋ Ca_2_^+^ + CO_3_^2-^; Cyronak et al., 2013). NO_3_^-^ uptake by heterotrophic bacteria can cause an increase in alkalinity and a reduction in DIC (Wolf-Gladrow et al., 2007).

### Muddy Sediment Gene Abundances

Similar patterns between the bacterial, archaeal, and cyanobacterial/chloroplast gene abundances (g^-1^ sediment) in all treatments were noticeable (**Figure 3**). Bacterial metabolism in aquatic environments is known to be regulated by temperature and resource availability (Degerman et al., 2013), and our results suggest that microbial growth was stimulated when seawater temperature increased by 4°C. This is consistent with other literature showing that cyanobacterial growth can be stimulated in response to increasing sea surface temperatures (e.g., Kanoshina et al., 2003; Sarmento et al., 2010). Model predictions have reiterated the potential impacts of increased seawater temperatures, leading to the suggestion that future environmental conditions may likely favor cyanobacterial blooms (Neumann et al., 2012; Tait et al., 2015a). However, the effect on cyanobacterial/chloroplast gene abundance was not replicated within our sequence data set; differences in relative abundance of cyanobacterial/chloroplast sequences were dominated by season (campaign) rather than treatment (results not shown). The different primers used for the analyses (V3-4 for qPCR and V1-3 for sequencing) may account for some of these differences observed.

Only the relative abundance of the Firmicutes was significantly impacted by the independent effects of elevated temperature (**Figure 6H**), and this group formed only a small proportion of the overall microbial community. This could be influenced by the availability of organic matter, and the initially high concentrations of NH_4_^+^ (**Table 1**) in the elevated temperature treatment, possibly indicative of relatively rapid diagenesis of organic matter in the sediment by bacterial metabolic processes. In all treatments, the average concentration of NH_4_^+^ decreased over the duration of the experiment, which is likely due to the oxidization of NH_4_^+^ to NO_3_^-^ by nitrifying bacteria which utilize the initially high availability of NH_4_^+^ from organic matter remineralization (Canfield et al., 2010). Up to 90% of dissolved inorganic nitrogen assimilation in estuaries is taken up in the form of NH_4_^+^ by heterotrophic bacteria (Middelburg and Nieuwenhuize, 2000). However, here there was no correlation between bacterial *amoA* gene abundance and nutrient production, and since archaeal *amoA* genes were barely detectable, it is very likely that the majority of NH_4_^+^ oxidation was not carried out by nitrifiers.

### Muddy Sediment Community Composition

Our findings from these relatively short-term experiments suggest that due to the natural variation in sediment pH (Silburn et al., 2017), the response of sediment microbial communities may be less evident under projected OA conditions compared to pelagic microbial communities (Joint et al., 2011). However, microbes have the capacity to rapidly acclimatize to changing environmental conditions (Hicks et al., 2017a) and therefore, results from short-term experiments may be misleading (Liu et al., 2010). Despite this, our findings did show that a distinct response from the sediment microbial communities over a longer time period, i.e., 28 days instead of 7 days (**Figures 3**, **4**). The nutrients data suggests the microorganisms in the elevated CO_2_ treatment were not limited by the availability of dissolved inorganic nitrogen in the overlying water.

Primary producers have shown to be affected by the increase in H^+^ concentrations leading to changes in the up-regulation (or down-regulation) of CO_2_-concentrating mechanisms (Hopkinson et al., 2011). Due to differences in the cyanobacterial and micro-algae starting community, we were unable to determine if this was linked to a change in community composition. The muddy sediments were dominated by several cyanobacterial species including the Oscillatoriales *Planktothricoides* and *Microcoleus*, Subsection II *Pleurocapsa* and Chroococcales *Synechococcus* (results not shown). In support of our results, studies have shown that growth may be negatively affected in various phytoplankton assemblages, e.g., diatoms (Gao et al., 2012b; Tait et al., 2015a); *Emiliania huxleyi* (Rokitta and Rost, 2012); and *Phaeocystis globosa* (Chen and Gao, 2011). Despite this, and in contrast to our results, positive effects of elevated CO_2_ on the growth of diatoms, phytoplankton, macroalgae, and cyanobacteria exist (see Gao et al., 2012a for a comprehensive list). This suggests the microbial response to environmental stressors will be species specific as well as stressor specific.

Tait et al. (2015b) showed similar results in response to a controlled sub-seabed CO_2_ leak where there was a decrease in abundance of microbial 16S rRNA genes (bacterial, archaeal, and primary producers) at the CO_2_ release site. This coincided with the highest measurements of DIC within the sediments, but may also have been related to the release of potentially toxic metals at this time point. Similarly, this study found a reduction in the abundance of microbial 16S rRNA genes, and this was linked to a change in microbial community composition but not to measurements of alpha diversity. However, previous studies using modest pH changes (up to 0.5 pH units) were also unable to detect any significant differences in alpha diversity between treatments (Tait et al., 2013, 2015a).

### Muddy Sediment Taxa Specific Responses

The mesocosm experiments reported within this study were replicated over time, meaning that there were different starting communities for the treatments. By using linear mixed-effects models, we were able to account for some of the variability evident in the starting communities by weighting for season (campaign). We have focused only on those dominant taxa showing significant differences across replicate experiments (Supplementary Table S5). Despite the differences in starting community, our data shows small but significant differences to community composition in the high CO_2_ treatments after 28 days incubation, with increases to the relative abundance of some taxa (Gammaproteobacteria, Deltaproteobacteria, Planctomycetacia, Actinobacteria, and Chloroflexi and decreases to the Cytophagia and Flavobacteria (**Figure 6**). The few studies that have examined the impact of elevated CO_2_ on surface sediment community composition have recorded different responses, with some showing no changes to community composition (Kerfahi et al., 2014). A few studies have shown major shifts in the community composition driven by increases to the relative abundance of microphytobenthos (Tait et al., 2015a), but the majority of studies have shown only modest changes to community composition (Tait et al., 2013, 2015b; Hassenrück et al., 2016). Tait et al. (2013, 2015b) showed increases to the relative abundance of 16S rRNA sequences affiliated to the Planctomycetacia, again of the *Rhodopirellula* sp., genera known to be influenced by pH (Buckley et al., 2006; Pollet et al., 2011), suggesting a preference of members of this taxa for lower pH environments. The increase in abundance of the Chloroflexi within the elevated CO_2_ treatment after 28 days incubation (**Figure 6G**) echoes the findings of Hassenrück et al. (2016). The taxa shown to shift in abundance within this study are predominantly heterotrophs and this suggests they may be more adaptable to an increase in CO_2_ concentration.

### Muddy Sediment Nitrogen Cycling Genes

This study saw a clear response in the AOB by day 28, with a decrease in abundance of the *amoA* genes, matching previous findings for a similar drop in pH (Tait et al., 2014). Microbial nitrification rates in the water column have been shown to be directly reduced by OA by up to 38% under experimental conditions (Beman et al., 2012), but sediment nitrification rates are not affected by OA (Kitidis et al., 2011; Braeckman et al., 2014; Watanabe et al., 2014). The preferred substrate for ammonia oxidation is thought to be NH_3_ (Suzuki et al., 1974; Stein et al., 1997). Elevated CO_2_ may indirectly affect nitrification by protonating NH_3_ to NH_4_^+^, thus a shift in the balance to the protonated form may be responsible for the reduction in *amoA* genes when pH is increased. Alternatively, a decrease in pH may impact ammonia monooxygenase activity (Ward, 1987). Nitrification is an important process to supply denitrifying bacteria with NO_3_^-^ required to break-down organic matter in the absence of oxygen (Koike and Sørensen, 1988). The reduction of *nirS* genes (**Figure 4C**) supports the reduction in abundance of nitrifying bacteria (measured as *amoA* genes) in the upper sediment layer. As seawater acidity increases due to OA, the removal of nitrogen in coastal regions may be affected if the rates of coupled nitrification–denitrification are impacted (Herbert, 1999). This is an important consideration for coastal areas, which are more vulnerable to experiencing localized eutrophication as well as experiencing larger natural variability.

Indirect impacts of OA on denitrifying microorganisms may also occur if microbial respiration increases under elevated seawater temperatures, although a reduction in oxygen penetration depth is likely to result in a greater surface area for denitrification to take place (Gehlen et al., 2011). However, in areas where rates of coupled nitrification–denitrification are high, shallowing of the oxic layer may impact on the production of NO_3_^-^ in the sediment and denitrifying bacteria will become more dependent on NO_3_^-^ diffusing into the sediment from the overlying water (Koop-Jakobsen and Giblin, 2010). Although analyses of changes to community composition indicated very few groups were impacted by the interactive and combined stressor effects of 750 ppm CO_2_ and 16°C (**Figure 6**), 16S rRNA (bacterial, archaeal, and cyanobacterial) gene abundance was significantly lower compared to ambient control conditions (**Figures 3A–C**, **4A,B**). Increasing water acidity coupled with increasing seawater temperatures is likely to put considerable pressure on microbial communities. Based on our findings, it is likely that microbial gene abundances will be reduced when elevated CO_2_ and elevated temperature are in combination, although this is likely to vary with microbial species. However, understanding the impacts reduced microbial growth will have on sediment processes and process rates is key to predicting long-term changes in coastal regions.

### Sandy Sediment Gene Abundance

In general, gene abundances were around one order of magnitude lower than those detected in the muddy sediment; a typical characteristic of permeable sediments (Rusch et al., 2001, 2003; Bühring et al., 2005) and nutrient concentrations were similar to concentrations measured in Braeckman et al. (2014). As expected, there was a considerable difference in nutrient dynamics between the two sediment types. Our results indicated that 16S rRNA gene abundance (archaeal and primary producers) were influenced by increased temperature after a week of incubation. In reality, the lower gene abundance was likely to be related to the community stabilizing under the new conditions (**Figures 5B,C**). The only indication that elevated temperature may have influenced metabolic processes was the flux of NO_X_ concentration in week 2 and higher concentrations of PO_4_^3-^ in weeks 3 and 4, although this was more likely linked to less absorption processes (e.g., pyrite formation) occurring in the sandy sediment (**Table 1**).

### Sandy Sediment Nitrogen Cycling Genes

Both archaeal *amoA* and bacterial *amoA* gene abundances appear to be influenced by elevated temperature. In culture, the effective maximum specific growth rate of AOB was found to increase with temperature in the range of 15–25°C (Antoniou et al., 1990), but much less is known of the influence of temperature on archaeal ammonia oxidizers other than their ability to grow at extremely low and high temperatures (Erguder et al., 2009). Increased abundance of archaeal *amoA* has been identified from a freshwater microcosm study when exposed to elevated temperature (Zeng et al., 2014). In contrast, Horak et al. (2013) found that there was no temperature sensitivity detected from a natural community dominated by archaea when they quantified ammonia-oxidation. The lack of sensitivity to increased temperature was thought to be due to low pH or trace metal concentrations at their study site. In our study, the increase in gene abundance is most likely due to a temperature-induced increase in the metabolic rate (Gillooly et al., 2001), particularly as these changes are most noticeable in the single stressor temperature only treatment.

Ammonia-oxidizing archaea are key players for nitrification and have shown a high affinity for ammonia in the marine environment, therefore changes in gene abundance of these microorganisms could impact not just process rates but also trophic interactions (Stahl and de la Torre, 2012). Permeable sediments and the associated microbial communities act as a biocatalyst for biogeochemical processes (Huettel et al., 1998; Jahnke et al., 2000; Reimersa et al., 2004). Due to advective flow through the sediment, organic matter and oxygen, as well as warmer water, is flushed through much deeper depths than in cohesive sediment (Hicks et al., 2017a), driving higher rates of microbial metabolism on the sediment particle surface and within the pore water (Rusch et al., 2001, 2003).

From the observed data it was evident that the treatment effects tended to be less pronounced in the sandy sediment samples (**Figure 5**) in comparison to the muddy sediment samples. The interactive effects of elevated CO_2_ and temperature had the strongest effect on microbial gene abundance. Archaeal 16S rRNA genes, bacterial *amoA*, archaeal *amoA* and bacterial *nirS* gene abundances were significantly lower, particularly seen in the archaeal *amoA* genes (**Figure 5B**). Comparing these results to the muddy sediment, the interactive effects of the two variables tended to induce a similar response. Lack of community composition analysis for the sandy sediment samples makes it more difficult to understand exactly what is happening to the community dynamics. Based on our present results, microbial communities in muddy estuarine sediments may be more impacted by changes in pH and increased seawater temperatures than sandy sediments. However, more information is needed to confidently determine how microbial communities in different sediments are likely to respond, acclimate and adapt in the face of global climate change. The interaction between elevated CO_2_ and elevated temperature on microbial communities remains uncertain due to the relatively small number of published studies investigating multiple drivers in marine sediments. Our research only begins to fill a major gap in OA research, and highlights the necessity of including other interacting factors that are influenced by human activities in future experimental design.

## Conclusion

This research suggests that there will be considerable independent and interactive effects of OA and increased temperature on sediment microbial communities, with differences between sediment types. Microbes closely related to the nitrogen cycle show variable responses to elevated CO_2_ and/or temperature (Kitidis et al., 2011), however, experiments that can quantify process rates concomitantly would be very valuable for more confident predictions. The present study was carried out over 28 days, and therefore, we were unable to identify if the microbial communities would eventually acclimatize to their new environmental conditions and reach a new “baseline” community after an initial rapid response. Information on acclimation and adaption in sediment microbial communities is, to our knowledge, very limited, and more emphasis should be given to long-term responses. Competition between benthic primary producers and microorganisms for nutrients is another possible scenario that should be further explored as benthic primary production can also be impacted by temperature, CO_2_ or the interactive of the two stressors (Cartaxana et al., 2015). Changes to key biogeochemical cycles in the sediment and the microbial communities that mediate these cycles will influence the dynamics of the marine environment. Integrated microbial and biogeochemical research is essential to fully explore the resilience of marine sediment microbial communities to climate change and how this will affect goods and services as major alterations to terrestrial and marine ecosystems occur now and in the coming years.

## Author Contributions

HS, NH, AO, and SW were responsible for the design and set-up of the experiments, which were performed by NH and AC. Sample analysis was carried out by KT, AC, and HP. Statistical analyses and interpretation were undertaken by AC and BdF-M. The manuscript was written by AC and NH with contributions from respective co-authors.

## Conflict of Interest Statement

The authors declare that the research was conducted in the absence of any commercial or financial relationships that could be construed as a potential conflict of interest.

## Footnotes

1 www.mrdnalab.com

2 https://www.bodc.ac.uk/data/published_data_library/catalogue/10.5285/559dfa45-285a-4323-e053-6c86abc0ed6c/
