# Supplementary material for: Marine Microbial Gene Abundance and Community Composition in Response to Ocean Acidification and Elevated Temperature in Two Contrasting Coastal Marine Sediments
Source: Front Microbiol. 2017 Aug 22;8:1599. doi: 10.3389/fmicb.2017.01599 (PMC5572232; doi:10.3389/fmicb.2017.01599)
Supplement: Supplementary file 1 [file Table_1.DOCX]

| **Table S1** Experimental design showing three replicates per environmental treatment. Four unique environmental conditions were used for each sediment type (mud and sand), with two CO_2_ levels (ambient: 380ppm; elevated: 750ppm) and two temperatures (ambient: 12°C; elevated: 16°C). | | |
| --- | --- | --- |
| **CO_2_ (ppm)** | **Temperature (°C)** | |
|  |  |  |
|  | **12** | **16** |
| **380** | **n=3** | **n=3** |
| **750** | **n=3** | **n=3** |
