# Supplementary material for: Marine Microbial Gene Abundance and Community Composition in Response to Ocean Acidification and Elevated Temperature in Two Contrasting Coastal Marine Sediments
Source: Front Microbiol. 2017 Aug 22;8:1599. doi: 10.3389/fmicb.2017.01599 (PMC5572232; doi:10.3389/fmicb.2017.01599)
Supplement: Supplementary file 2 [file Table_2.DOCX]

| **Table S2** Summary table of primers (16S rRNA and functional genes) used for PCR and q-PCR amplification | | | | | |
| --- | --- | --- | --- | --- | --- |
| **Target Organism** | **Name of primer** | **Sequence of primer**  **(5’ - 3’)** | **Primer concent-ration** | **Cycling conditions** | **Reference** |
| Universal Bacterial 16S rRNA genes | Bact 1369F  Prok 1492R | cggtgaatacgttcycgg  ggwtaccttgttacgactt | 900 nM  300 nM | 95˚C for 5 min  Then 40 cycles of 95˚C for 15 sec and 60˚C for 1 min. | [Suzuki *et al.* (2000](#_ENREF_97)) |
| Universal Archaeal 16S rRNA genes | Parch519f  ARC915r | cagccgccgcggtaa  gtgctcccccgccaattcct | 300 nM  300 nM | 95˚C for 5 min  Then 40 cycles of 95˚C for 15 sec and 63˚C for 1 min. | [Ovreås *et al.* (1997](#_ENREF_76)) |
| Universal Cyanobacterial/ Chloroplast 16S rRNA genes | CYA-359F  CYA-781R | GGGGAATYTTCCGCAATGGG  GACTACWGGGGTATCTAATCW | 200 nM  200 nM | 95˚C for 5 min  Then 40 cycles of 95˚C for 15 sec and 58˚C for 1 min. | [Nübel *et al.* (1997](#_ENREF_73)) |
| Bacterial ammonia monooxygenase (*amoA*) genes | amoA1F  amoA2R | GGGGHTTYTACTGGTGGT  CCCCTCKGSAAAGCCTTCTTC | 900 nM  900 nM | 95˚C for 5 min  Then 40 cycles of 95˚C for 15 sec and 61.5˚C for 1 min. | [Stephen *et al.* (1996](#_ENREF_94))  [Hornek *et al.* (2006](#_ENREF_48)) |
| Archaeal ammonia monooxygenase (*amoA*) genes | CamoA-23F  CamoA-616R | ATGGTCTGGYTWAGACG  GCCATCCABCKRTANGTCCA | 300 nM  300 nM | 95˚C for 5 min  Then 40 cycles of 95˚C for 15 sec and 58.5˚C for 1 min. | [Tourna *et al.* (2008](#_ENREF_102))  [Nicol *et al.* (2008](#_ENREF_72)) |
| Archaeal ammonia monooxygenase (*amoA*) genes | Arch-amoA-for  Arch-amoA-rev | CTGAYTGGGCYTGGACATC  TTCTTCTTTGTTGCCCAGTA | 300 nM  300 nM | 95˚C for 5 min  Then 40 cycles of 95˚C for 15 sec and 58.5˚C for 1 min. | Wutcher et al. (2006) |
| Archaeal ammonia monooxygenase (*amoA*) genes | Arch-amoA  Arch-amoA | STAATGGTCTGGCTTAGACG  GCGGCCATCCATCTGTATGT | 300 nM  300 nM | 95˚C for 5 min  Then 40 cycles of 95˚C for 15 sec and 56˚C for 1 min. | Francis et al. (2005) |
| Bacterial nitrite reductase (*nirS*) genes | nirS1F  nirS3R | CCTAYTGGCCGCCRCART  GCCGCCGTCRTGVAGGAA | 900 nM  900 nM | 95˚C for 5 min  Then 40 cycles of 95˚C for 15 sec and 62˚C for 1 min. | [Braker *et al.* (1998](#_ENREF_10)) |
