# Supplementary material for: Marine Microbial Gene Abundance and Community Composition in Response to Ocean Acidification and Elevated Temperature in Two Contrasting Coastal Marine Sediments
Source: Front Microbiol. 2017 Aug 22;8:1599. doi: 10.3389/fmicb.2017.01599 (PMC5572232; doi:10.3389/fmicb.2017.01599)
Supplement: Supplementary file 3 [file Table_3.DOCX]

| **Table S3** q-PCR and RT – q-PCR statistics. For the standard curve for each target gene, the slope, y intercept, the efficiency of amplification and co-efficient of determination (r^2^) were determined. | | | | |
| --- | --- | --- | --- | --- |
| Gene | Slope | Intercept | Efficiency | r^2^ |
| Bacterial 16S rRNA | -3.33 | 35.92 | 99.5 % | 0.998 |
| Archaeal 16S rRNA | -3.25 | 34.12 | 103.3 % | 0.993 |
| Cyanobacterial 16S rRNA | -3.29 | 37.41 | 101.3 % | 0.997 |
| Bacterial *amoA* | -3.47 | 36.62 | 94.2 % | 0.997 |
| Archaeal *amoA* | -3.21 | 33.74 | 104.9 % | 0.997 |
| Bacterial *nirS* | -3.3 | 34.44 | 100.9 % | 0.998 |
