# Supplementary material for: Marine Microbial Gene Abundance and Community Composition in Response to Ocean Acidification and Elevated Temperature in Two Contrasting Coastal Marine Sediments
Source: Front Microbiol. 2017 Aug 22;8:1599. doi: 10.3389/fmicb.2017.01599 (PMC5572232; doi:10.3389/fmicb.2017.01599)
Supplement: Supplementary file 4 [file Table_4.DOCX]

| **Table S4** Summary of Linear Mixed-Effects Model output describing effects of elevated CO_2_ and/or temperature on microbial gene abundances in muddy and sandy sediment and with varying sediment depth. Independent and interactive terms are presented with significant results indicated in bold. | | | | | | | |
| --- | --- | --- | --- | --- | --- | --- | --- |
| **Gene** | **Sediment**  **type** | **Depth (cm)** | **Data transformation** | **Linear Mixed Effects Model** | | | |
|  |  |  |  | **Factor** | **Coefficient** | ***t*** | ***p*** |
| Bacterial 16S rRNA genes | Mud | 0-0.5 | Square root | CO_2_^750^  Temp^16^  T^7^  T^28^  T^28^ x Temp^16^  T^28^ x CO_2_^750^  Temp^16^ x CO_2_^750^ | 5148.68  -997.29  5527.95  1102.78  12668.24  -24451.36  -10145.76 | 1.07  -0.21  1.12  0.22  2.37  -4.62  -2.47 | 0.28  0.83  0.26  0.82  **0.01**  **<0.0001**  **0.01** |
| Archaeal 16S rRNA genes | Mud | 0-0.5 | Log_10_ | CO_2_^750^  Temp^16^  T^7^  T^28^  T^28^ x Temp^16^  T^28^ x CO_2_^750^ | 0.16  -0.09  -0.14  0.12  0.56  -0.94 | 1.07  -0.65  -0.93  0.79  3.31  -5.64 | 0.28  0.51  0.35  0.42  **<0.001**  **<0.0001** |
| Cyanobacterial/ chloroplast 16S rRNA genes | Mud | 0-0.5 | Square root | CO_2_^750^  Temp^16^  T^7^  T^28^  T^28^ x Temp^16^  T^28^ x CO_2_^750^  Temp^16^ x CO_2_^750^ | 10942.60  -3926.86  12342.89  1588.79  29034.08  -41180.69  -15099.18 | 1.26  -0.46  1.37  0.17  2.99  -4.28  -2.02 | 0.2  0.6  0.1  0.8  **0.002**  **<0.0001**  **0.04** |
| Bacterial *amoA* genes | Mud | 0-0.5 | Log_10_ | CO_2_^750^  Temp^16^  T^7^  T^28^  T^28^ x CO_2_^750^ | -0.16  -0.34  -0.11  0.15  -0.45 | -1.01  -4.09  -0.79  1.02  -2.11 | 0.3  **<0.0001**  0.43  0.30  **0.03** |
| Bacterial 16S rRNA genes | Mud | 0.5-2.5 | Square root | CO_2_^750^  Temp^16^  T^7^  T^28^  T^28^ x Temp^16^  T^28^ x CO_2_^750^  Temp^16^ x CO_2_^750^ | 10040.69  2441.60  6834.07  7320.04  11197.12  -17537.40  -14278.86 | 2.67  0.66  1.76  1.88  2.47  -3.87  -3.89 | **<0.05**  0.50  **<0.05**  **0.05**  **0.01**  **<0.001**  **<0.0001** |
| Archaeal 16S rRNA genes | Mud | 0.5-2.5 | Square root | CO_2_^750^  Temp^16^  T^7^  T^28^  T^28^ x Temp^16^  T^28^ x CO_2_^750^  Temp^16^ x CO_2_^750^ | 2535.37  984.21  259.73  3577.04  3365.28  -5165.16  -3709.12 | 3.71  1.47  0.36  5.05  4.08  -6.27  -5.56 | **<0.001**  0.13  0.71  **<0.0001**  **<0.0001**  **<0.0001**  **<0.0001** |
| Bacterial *nirS* genes | Mud | 0.5-2.5 | Square root | CO_2_^750^  Temp^16^  T^7^  T^28^  T^28^ x Temp^16^  T^28^ x CO_2_^750^ | 1875.69  -2726.12  610.58  1271.81  5104.73  -6553.78 | 1.99  -2.90  0.52  1.09  3.79  -4.87 | **0.04**  **<0.01**  0.59  0.27  **0.001**  **<0.0001** |
| Bacterial 16S rRNA genes | Sand | 0-1 | Square root | T^7^  T^28^ | 174.64  1324.41 | 0.43  3.29 | 0.66  **<0.001** |
| Archaeal 16S rRNA genes | Sand | 0-1 | Log_10_ | CO_2_^750^  Temp^16^  T^7^  T^28^  T^7^ x Temp^16^  Temp^16^ x CO_2_^750^ | 0.10  0.12  0.08  0.19  -0.21  -0.31 | 1.94  1.74  1.29  3.08  -2.37  -4.26 | **0.05**  0.08  0.19  **0.002**  **0.01**  **<0.0001** |
| Cyanobacterial/ chloroplast 16S rRNA genes | Sand | 0-1 | Log_10_ | CO_2_^750^  Temp^16^  T^7^  T^28^  T^7^ x Temp^16^ | -0.002  0.07  -0.02  0.02  -0.17 | 0.07  1.16  -0.41  0.40  -2.02 | 0.94  0.24  0.68  0.68  **0.04** |
| Bacterial *nirS* genes | Sand | 0-1 | Log_10_ | CO_2_^750^  Temp^16^  T^7^  T^28^  Temp^16^ x CO_2_^750^ | 0.02  0.04  -0.14  -0.02  -0.12 | 0.71  1.58  -5.27  -1.07  -2.82 | 0.47  0.11  **<0.0001**  0.28  **0.004** |
| Bacterial *amoA* genes | Sand | 0-1 | Log_10_ | CO_2_^750^  Temp^16^  T^7^  T^28^  Temp^16^ x CO_2_^750^ | 0.11  0.14  -0.008  0.07  -0.27 | 3.57  4.27  -0.28  2.75  -5.80 | **<0.001**  **<0.0001**  0.77  **0.005**  **<0.0001** |
| Archaeal *amoA* genes | Sand | 0-1 | Log_10_ | CO_2_^750^  Temp^16^  T^7^  T^28^  Temp^16^ x CO_2_^750^ | 0.08  0.13  -0.06  -0.12  -0.23 | 1.22  1.89  -1.01  -2.05  -2.34 | 0.22  **0.05**  0.30  **0.03**  **0.01** |
