# Supplementary material for: Marine Microbial Gene Abundance and Community Composition in Response to Ocean Acidification and Elevated Temperature in Two Contrasting Coastal Marine Sediments
Source: Front Microbiol. 2017 Aug 22;8:1599. doi: 10.3389/fmicb.2017.01599 (PMC5572232; doi:10.3389/fmicb.2017.01599)
Supplement: Supplementary file 5 [file Table_5.DOCX]

| **Table S5** Summary of Tukey’s Tests from Linear Mixed-Effects Models describing effects of elevated CO_2_ and/or temperature on relative sequence abundance (%) in muddy sediment. All results presented show significant pairwise comparisons between different treatments and time points (T). The Alpha Diversity results are also given. Models that could not be run due to (a) high seasonal variation are indicted by SV; (b) significant differences between control treatments are indicated by DCT; (c) no significant effects are shown as NS; (d) other factors affecting changes are shown as OF; and (e) bad model fit are shown as BMF. |
| --- |

|  |  |  | **Tukey’s Test**  **______________________________________________** | | |
| --- | --- | --- | --- | --- | --- |
| **Phylum** | **Class** | **Relative Sequence Abundance (%)** | **Factors** | ***z*** | ***p*** |
| *Proteobacteria* |  | 49.07 |  |  | OF |
|  | *Gammaproteobacteria* | 24.5 | CO_2_^750^, T^28^  CO_2_^750^, T^7^, T^28^  CO_2_^750^, Temp^16^, T^28^ | 3.606  4.793  3.527 | 0.01  <0.01  0.02 |
|  | *Deltaproteobacteria* | 14.5 | CO_2_^750^, T^28^ | 3.536 | 0.01 |
|  | *Alphaproteobacteria* | 6.56 |  |  | SV/DCT |
|  | *Epsilonproteobacteria* | 1.63 |  |  | SV/DCT |
| *Bacteriodetes* |  | 32.70 |  |  | DCT |
|  | *Cytophagia* | 18.66 | CO_2_^750^, T^28^  CO_2_^750^, CO_2_^750^ x Temp^16^, T^7^  CO_2_^750^, Temp^16^, T^28^ | -5.025  -3.413  -3.526 | <0.01  0.03  0.02 |
|  | *Flavobacteria* | 10.65 | CO_2_^750^, T^28^  CO_2_^750^ x Temp^16^, T^28^  CO_2_^750^, T^7^, T^28^  CO_2_^750^, Temp^16^, T^28^ | -3.509  -3.313  -4.398  -3.242 | 0.01  0.03  <0.01  0.02 |
|  | *Sphingobacteria* | 2.5 |  |  | NS |
| *Verrucomicrobia* |  | 3.01 |  |  | SV |
| *Euryarchaeota* |  | 2.89 |  |  | OF |
| *Planctomycetes* |  | 2.80 | CO_2_^750^, T^28^  CO_2_^750^, T^7^, T^28^  CO_2_^750^, Temp^16^, T^28^ | 7.274  8.115  5.330 | <0.001  <0.001  <0.001 |
|  | *Planctomycetacia* | 2.57 | CO_2_^750^ x Temp16, T^7^  CO_2_^750^, T^7^, T^28^  CO_2_^750^, Temp^16^, T^28^ | -3.606  4.793  3.527 | 0.01  <0.01  0.02 |
| *Actinobacteria* |  | 1.93 | CO_2_^750^, T^28^  CO_2_^750^ x Temp^16^, T^28^  CO_2_^750^, Temp^16^, T^28^ | 6.590  3.165  5.458 | <0.001  0.05  <0.001 |
| *Chloroflexi* |  | 1.35 | Temp^16^, T^7^  CO_2_^750^, T^28^  CO_2_^750^ x Temp^16^, T^7^  CO_2_^750^, T^7^, T^28^  CO_2_^750^, Temp^16^, T^28^  CO_2_^750^ x Temp^16^, CO_2_^750^, T^28^ | 3.337  5.833  3.498  5.383  6.043  4.160 | 0.03  <0.01  0.02  <0.01  <0.01  0.01 |
| *Firmicutes* |  | 1.05 | Temp^16^, T^28^  CO_2_^750^ X Temp^16^, Temp^16^, T^28^ | 3.456  -3.613 | 0.02  0.01 |
| WS3 (candidate division) |  | 1.03 |  |  | BMF |
| **Alpha Diversity** | | | | | |
| Species Richness |  |  |  |  | NS |
| Pielou Evenness |  |  |  |  | NS |
| Shannon Diversity |  |  |  |  | NS/BMF |
