# Supplementary figures and images for: Marine Microbial Gene Abundance and Community Composition in Response to Ocean Acidification and Elevated Temperature in Two Contrasting Coastal Marine Sediments
Source: Front Microbiol. 2017 Aug 22;8:1599. doi: 10.3389/fmicb.2017.01599 (PMC5572232; doi:10.3389/fmicb.2017.01599)

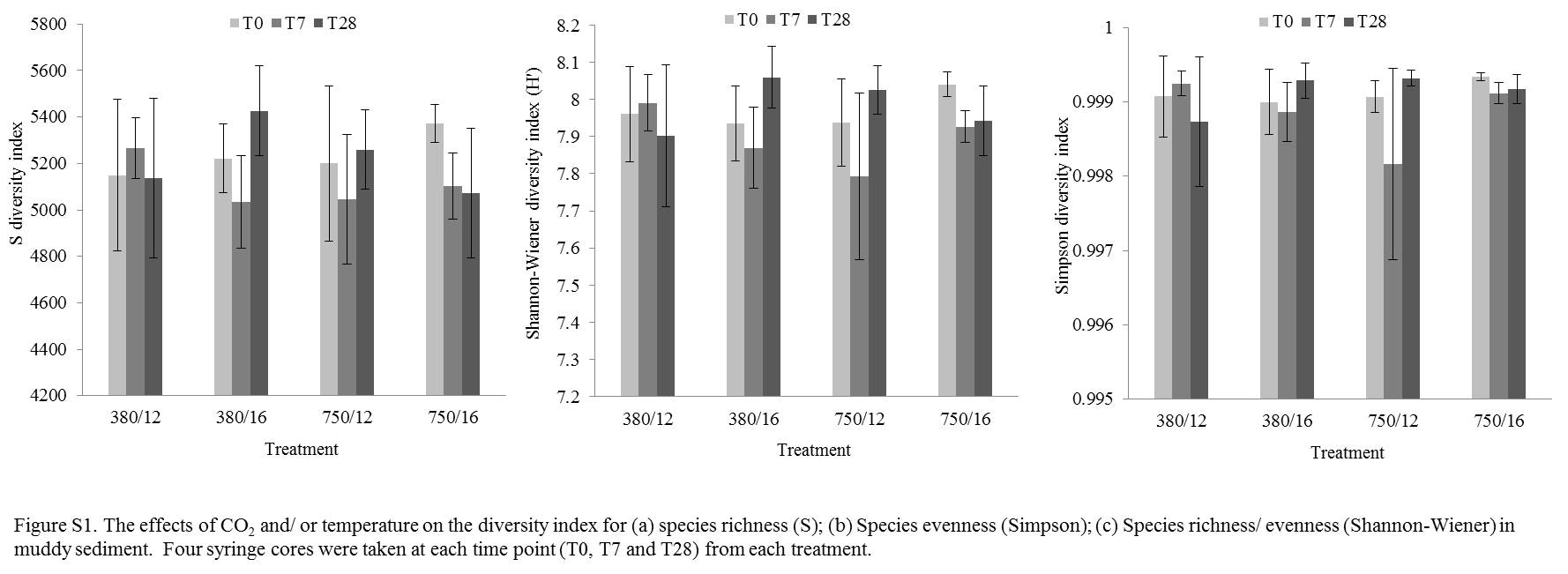

Supplement: Supplementary file 6 [file Image_1.JPEG]
